# Axillary-pouch capsular thinning after ultrasound-guided hydrodilation in post-stroke adhesive capsulitis: a longitudinal observational study

**DOI:** 10.1007/s40477-026-01208-x

**Published:** 2026-08-05

**Authors:** Filippo Cotellessa, Luca Puce, Salvatore Massimo Stella, Riccardo Picasso, Vittorio Anfossi, Laura Mori, Maria Cesarina May, Matteo Formica, Carlo Trompetto, William Campanella

**Affiliations:** 1 Department of Neuroscience, Rehabilitation, Ophthalmology, Genetics, Maternal and Child Health (DINOGMI), University of Genoa, 16132 Genoa, Italy; 2 IRCCS Azienda Ospedaliera Metropolitana, 16132 Genoa, Italy; 3 Department of Clinical and Experimental Medicine, Post-Graduate School of Sports Medicine, Santa Chiara University Hospital, University of Pisa, Via Roma 67, 56100 Pisa, Italy; 4 Department of Integrated Surgical Diagnostic Sciences (DISC), Orthopedic Clinic, University of Genoa, 16132 Genoa, Italy

**Keywords:** Stroke, Adhesive capsulitis, Hydrodilation, Ultrasonography, Axillary pouch

## Abstract

**Purpose:**

To characterize longitudinal changes in axillary-pouch capsular thickness and clinical outcomes after ultrasound-guided hydrodilation with corticosteroid injection in patients with post-stroke adhesive capsulitis, and to examine associations between structural and clinical changes.

**Methods:**

In this longitudinal observational study, 20 patients underwent clinical and ultrasonographic assessment before the first procedure (T0) and at follow-up (T1), separated by a median of 5.7 months. All participants also received conventional neurorehabilitation. Paired changes in pain measured using the Numeric Rating Scale (NRS), range of motion (ROM), strength, muscle tone, disability, quality of life, and capsular thickness were assessed using Wilcoxon signed-rank or exact McNemar tests. Associations between within-participant changes were explored using Spearman correlations.

**Results:**

Median movement-evoked pain decreased by 4 NRS points during flexion and abduction and by 5 points during external rotation (all *p* < 0.001). Active and passive ROM improved across all assessed movements (all *p* ≤ 0.001). Median affected-side capsular thickness decreased from 3.7 to 2.45 mm, with a median within-participant reduction of 1.4 mm (*p* < 0.001). The proportion meeting the ultrasonographic criteria for adhesive capsulitis decreased from 20/20 at T0 to 4/20 at T1. Capsular-thickness changes were not significantly associated with changes in any clinical outcome (all |ρ|≤ 0.35; all *p* ≥ 0.13).

**Conclusion:**

In this uncontrolled cohort, hydrodilation with corticosteroid injection, delivered alongside neurorehabilitation, was followed by reduced movement-evoked pain, improved shoulder mobility, and axillary-pouch capsular thinning. Capsular thickness should be interpreted as a complementary structural marker rather than a surrogate for clinical recovery.

## Introduction

Shoulder pain is a common complication of stroke and may significantly interfere with rehabilitation, activities of daily living, and quality of life [1–3]. Its etiology is multifactorial and involves overlapping neurological and musculoskeletal mechanisms, including muscle weakness, altered muscle tone and motor control, sensory impairment, abnormal scapulohumeral mechanics, and peripheral soft-tissue lesions [4,5]. Among these conditions, adhesive capsulitis represents an important and potentially modifiable cause of pain and restricted passive shoulder mobility [6].

Adhesive capsulitis is characterized by synovial inflammation, capsular fibrosis, and reduced glenohumeral joint capacity, resulting in a progressive limitation of both active and passive range of motion (ROM), particularly external rotation [7–10]. After stroke, capsular restriction may add to the underlying neurological impairment, further limiting passive shoulder mobility and hindering rehabilitation [1,11,12]. Diagnosis remains primarily clinical, although imaging can support diagnostic assessment and characterize capsular abnormalities. Magnetic resonance imaging can demonstrate capsular thickening and inflammatory changes [13], whereas ultrasound can identify thickening of the axillary-pouch capsule and concomitant shoulder abnormalities. An axillary-pouch thickness greater than 4 mm or marked asymmetry relative to the contralateral shoulder has been proposed as a useful diagnostic feature [14–17]. Owing to its accessibility, repeatability, and suitability for procedural guidance, ultrasound may also provide an objective tool for monitoring structural capsular changes over time [15].

Hydrodilation consists of intra-articular fluid distension, generally combined with corticosteroid and local anesthetic administration, with the aim of reducing capsular restriction and inflammation. Studies conducted predominantly in patients with primary adhesive capsulitis have reported short-term improvements in pain and ROM. However, comparative evidence remains heterogeneous and has not consistently demonstrated the superiority of hydrodilation over other conservative treatments [18–21]. A recent study conducted in patients with primary adhesive capsulitis also documented a reduction in capsular thickness after ultrasound-guided hydrodilation [22]. However, it remains unclear whether this reduction reflects clinical improvement. Moreover, it is not known whether similar structural changes occur in patients with post-stroke adhesive capsulitis, for whom evidence regarding the use of hydrodilation remains limited.

The relationship between structural changes and clinical recovery may be particularly complex after stroke, because pain, mobility, and upper-limb function are influenced not only by capsular restriction but also by neurological recovery, muscle weakness, altered tone and motor control, sensory impairment, and concurrent rehabilitation. Symptomatic and functional improvement may therefore not occur in parallel with capsular thinning. Accordingly, the primary aim of this study was to quantify longitudinal changes in axillary-pouch capsular thickness following ultrasound-guided hydrodilation with intra-articular corticosteroid injection in patients with post-stroke adhesive capsulitis. Secondary aims were to evaluate changes in clinical outcomes and to explore whether the magnitude of capsular thinning was associated with individual changes in pain, ROM, muscle strength, muscle tone, disability, and quality of life.

## Materials and methods

### Participants

Patients were consecutively recruited from the Neurorehabilitation Unit of a tertiary university hospital. Eligible participants were adults with a first-ever imaging-confirmed ischemic or hemorrhagic stroke, contralateral hemiparesis, and pain in the paretic shoulder. Exclusion criteria were Glasgow Coma Scale score < 8, cognitive or behavioral impairment preventing assessment, and pre-stroke shoulder pain or dysfunction. Adhesive capsulitis was clinically defined by > 50% loss of passive external rotation versus the contralateral side, together with at least 50% loss of passive flexion and abduction and no explanatory radiographic abnormality.

### Study design

This longitudinal observational study included baseline clinical and ultrasonographic assessment before the first hydrodilation (T0) and follow-up assessment after the last procedure (T1). Patients with clinically and ultrasonographically confirmed adhesive capsulitis were offered ultrasound-guided glenohumeral hydrodilation with corticosteroid injection. All participants concurrently received conventional physiotherapy tailored to their neurological impairment.

The study was approved by the competent institutional ethics committee (details blinded for peer review) and was conducted in accordance with the Declaration of Helsinki. All participants provided written informed consent before study participation.

### Clinical assessment

Active and passive shoulder flexion, abduction, and external rotation were measured using a handheld goniometer. Strength in the same movement planes was graded using the Medical Research Council scale, and muscle tone of the corresponding antagonist groups was graded using the Modified Ashworth Scale, with grade 1 + converted to 1.5 for analysis [23,24]. Pain intensity was rated on an 11-point Numeric Rating Scale, ranging from 0 (“no pain”) to 10 (“worst imaginable pain”), at rest and during passive shoulder flexion, abduction, and external rotation. Disability was assessed using the Disabilities of the Arm, Shoulder and Hand (DASH) questionnaire. Health-related quality of life was assessed using the 12-item Short Form Health Survey (SF-12), expressed as Physical Component Summary (PCS) and Mental Component Summary (MCS) scores [25,26].

### Ultrasound assessment

Two experienced physiatrists jointly examined both shoulders using an Esaote MyLab XPro75 system equipped with 4–15-MHz and 3–11-MHz linear transducers. The rotator cuff and long-head biceps tendons were assessed for tendinopathy and tearing according to standard sonographic criteria [27]. Tendinopathy and tear scores represented the number of involved tendons. Subacromial-subdeltoid bursitis and long-head biceps tendon sheath effusion were defined as fluid distension > 2 mm, whereas glenohumeral joint effusion was defined as a distance > 2 mm between the deep infraspinatus fibers and the posterior glenoid labrum [28].

**Fig. 1:**
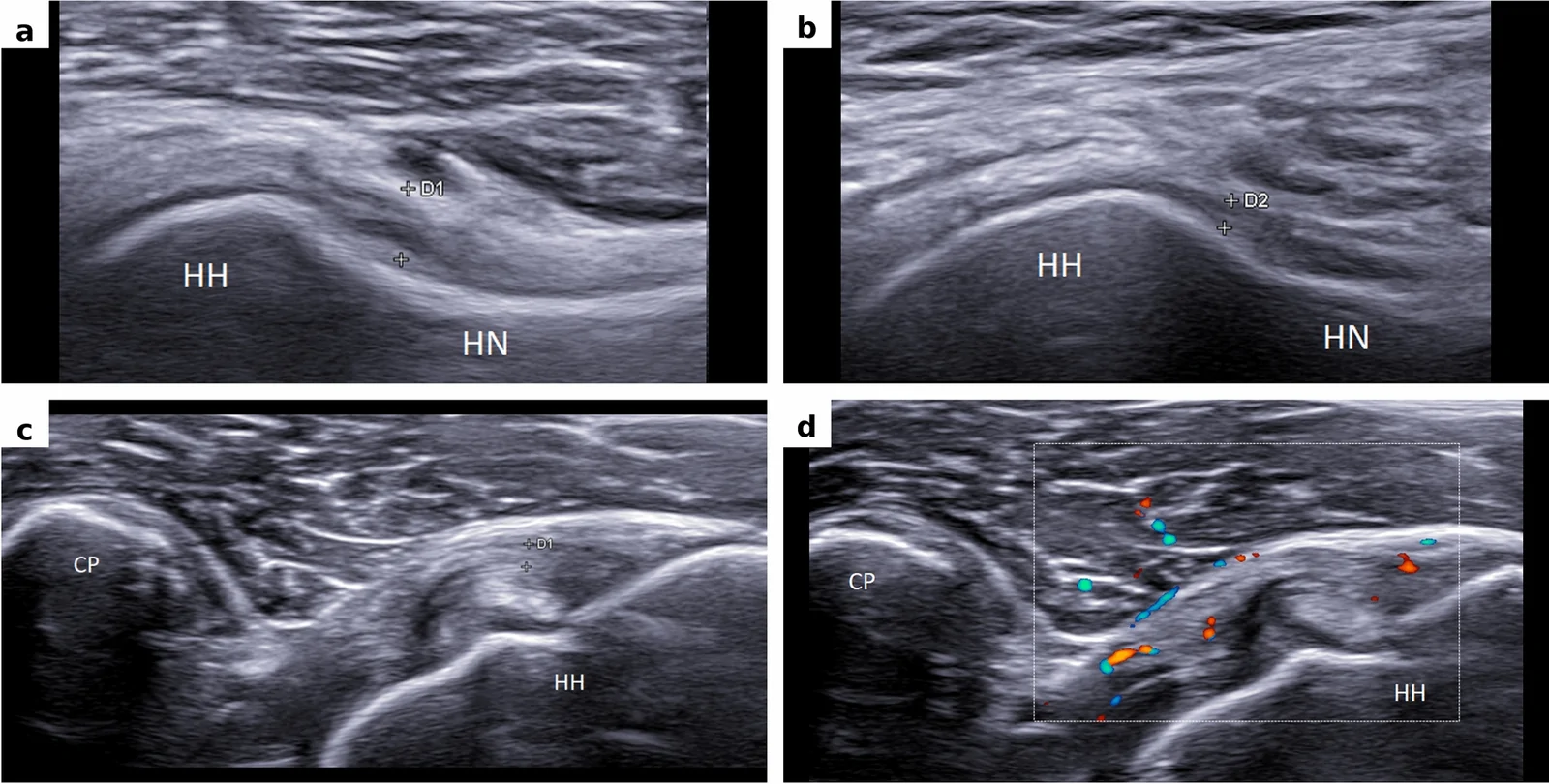
Ultrasonographic findings in adhesive capsulitis. **a** Axillary-pouch capsule before treatment (T0), showing inferior capsular thickening. **b** Corresponding follow-up image (T1), showing reduced capsular thickness. D1 and D2 indicate capsular measurements over the humeral head and along the humeral neck, respectively. c Thickening of the coracohumeral ligament. d Power Doppler hypervascularity at the rotator interval involving the coracohumeral ligament and biceps pulley. *CP* coracoid process, *HH* humeral head, *HN* humeral neck

Inferior capsular thickness was measured at the axillary pouch with the arm abducted 70–90° in neutral rotation. The ultrasonographic criteria for adhesive capsulitis were an affected-side capsular thickness > 4 mm or a > 60% difference relative to the contralateral side [14,16]. Representative images of axillary-pouch capsular thickness at T0 and T1 are shown in Fig. 1a and b. Additional ultrasonographic findings, including coracohumeral ligament thickening and Power Doppler hypervascularity of the rotator interval, were recorded as present or absent and are illustrated in Fig. 1c and d.

Reliability of capsular-thickness measurements was assessed using 40 stored ultrasound images. For inter-observer reliability, two experienced observers independently measured each image while blinded to the other observer’s measurements. For intra-observer reliability, one observer repeated the measurements after a two-week interval while blinded to the original values.

### Hydrodilation procedure

**Fig. 2:**
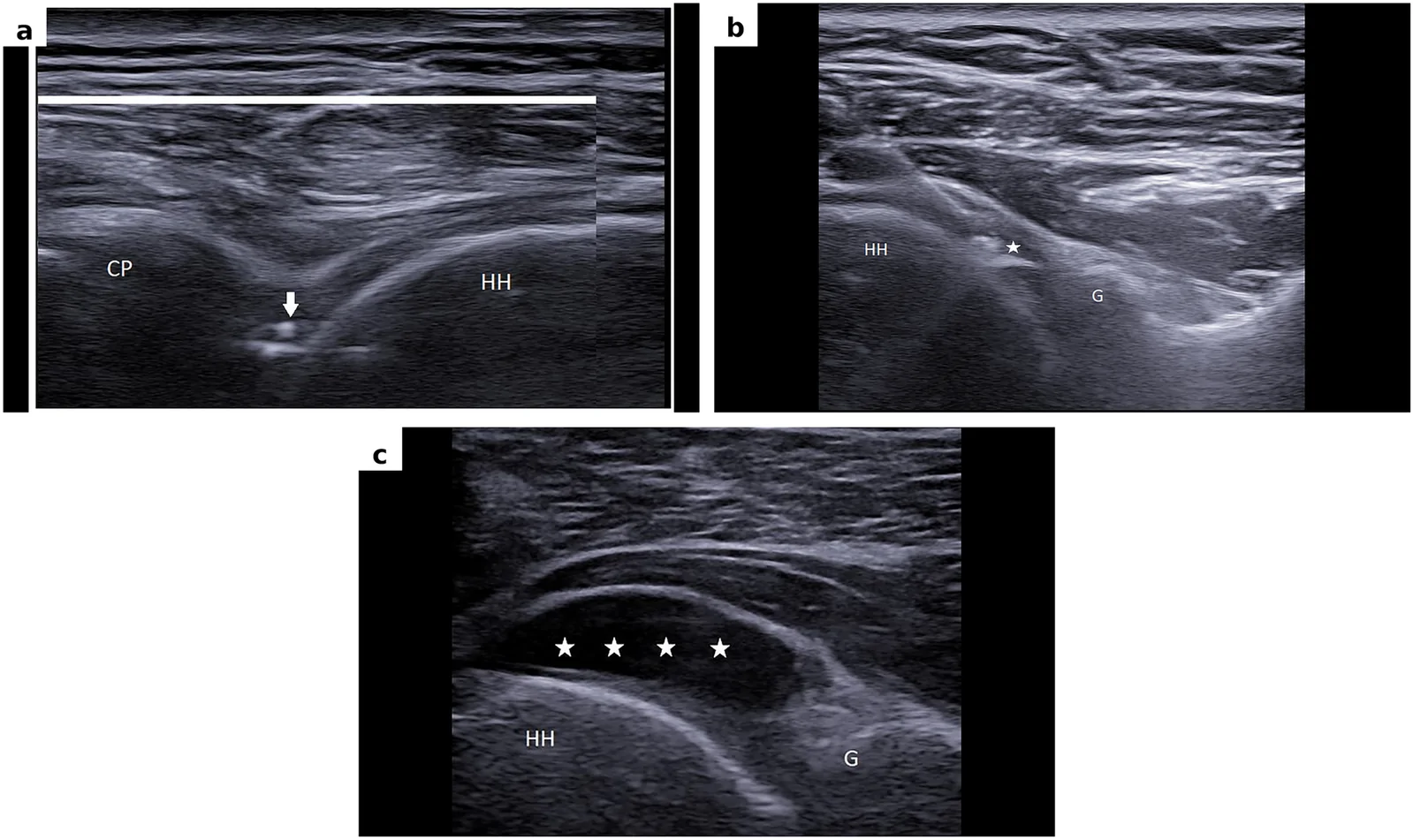
Ultrasound-guided hydrodilation procedure. **a** Anterior glenohumeral approach using an out-of-plane technique; the arrow indicates the intra-articular needle tip. **b** Posterior approach using an in-plane technique; the star indicates the intra-articular needle tip. **c** Distension of the posterior glenohumeral recess after fluid injection, confirming intra-articular distribution. *CP* coracoid process, *G* glenoid, *HH* humeral head

Under sterile real-time ultrasound guidance, the glenohumeral joint was accessed via either an anterior out-of-plane approach or a posterior in-plane approach using a 21–22-gauge, 40-mm needle (Fig. 2a and b).

After confirmation of intra-articular needle placement, 3–5 mL of 2% lidocaine and 1 mL of triamcinolone acetonide (40 mg/mL) were injected, followed by high-volume hydrodistension with 30–50 mL of normal saline. The final volume was individualized according to capsular compliance, injection resistance, and patient tolerance. Successful capsular distension was confirmed by real-time ultrasonographic visualization of expansion of the glenohumeral joint recesses and adequate intra-articular distribution of the injected fluid (Fig. 2c).

The indication for an additional procedure was established at the 15-day clinical reassessment when the response was considered insufficient, defined as < 50% ROM recovery across all movement planes or movement-evoked pain > 4/10. A maximum of three procedures was permitted. Patients were monitored for 30 min after each procedure. Follow-up clinical and ultrasonographic assessment was performed after the final procedure.

### Statistical analysis

Age was summarized as mean, standard deviation, and range. Other continuous and ordinal variables were summarized as medians and interquartile ranges, and categorical variables as counts and percentages. Paired continuous or ordinal outcomes were compared using the Wilcoxon signed-rank test, with effect size r calculated from the standardized test statistic. Paired binary ultrasound outcomes were compared using the exact McNemar test.

Associations between within-participant changes in affected-side capsular thickness and clinical outcomes were explored using Spearman rank correlations. Inter- and intra-observer reliability of capsular-thickness measurements was assessed using absolute-agreement, single-measure intraclass correlation coefficients with 95% confidence intervals. Measurement error was quantified using the standard error of measurement and the 95% minimal detectable change. Diagnostic agreement for adhesive capsulitis classification was assessed using Cohen’s kappa and percentage agreement. Tests were two-tailed with *p* < 0.05. Because the correlation analyses were exploratory, no adjustment for multiple comparisons was applied. Analyses were performed in Python in a Jupyter Notebook environment.

## Results

### Participant characteristics and clinical outcomes

Of 40 patients screened, 25 met the study eligibility criteria and underwent baseline assessment. Of these, 20 completed follow-up and were included in the paired analyses. Among the 20 participants included in the longitudinal analysis, mean age at T0 was 62.6 ± 12.9 years (range, 31.8–84.9), 9/20 (45.0%) were male, 10/20 (50.0%) had ischemic stroke, and 10/20 (50.0%) had left-sided paresis. All participants met at least one of the prespecified ultrasonographic criteria for adhesive capsulitis at baseline: 10/20 (50.0%) had an affected-side capsular thickness greater than 4 mm, and 18/20 (90.0%) showed a greater than 60% increase relative to the contralateral side. Ten participants underwent one hydrodilation procedure, seven underwent two procedures, and three underwent three procedures. The median interval between T0 and T1 was 172 days (IQR, 155.8–204.3; range, 78–507), corresponding to 5.7 months (IQR, 5.1–6.7; range, 2.6–16.7) (Table 1).

**Table 1:** Baseline, procedural, and follow-up characteristics of the study population

Characteristic	Baseline cohort (*n* = 25)	Paired-analysis cohort (*n* = 20)
Age at T0, years, mean ± SD	63.6 ± 12.3	62.6 ± 12.9
Age range, years	31.8–84.9	31.8–84.9
Male sex, n (%)	13 (52.0)	9 (45.0)
Female sex, n (%)	12 (48.0)	11 (55.0)
Ischemic stroke, n (%)	13 (52.0)	10 (50.0)
Hemorrhagic stroke, n (%)	12 (48.0)	10 (50.0)
Left-sided paresis, n (%)	14 (56.0)	10 (50.0)
Right-sided paresis, n (%)	11 (44.0)	10 (50.0)
Affected-side capsular thickness > 4 mm, n (%)	15 (60.0)	10 (50.0)
Capsular asymmetry > 60%, n/N (%)	22/24 (91.7)^a^	18/20 (90.0)
At least one ultrasonographic criterion, n (%)	25 (100.0)	20 (100.0)
One hydrodilation procedure, n (%)	12 (48.0)	10 (50.0)
Two hydrodilation procedures, n (%)	10 (40.0)	7 (35.0)
Three hydrodilation procedures, n (%)	3 (12.0)	3 (15.0)
T0–T1 interval, days, median (IQR)	—	172 (155.8–204.3)
T0–T1 interval, days, range	—	78–507
T0–T1 interval, months, median (IQR)	—	5.7 (5.1–6.7)
T0–T1 interval, months, range	—	2.6–16.7

*IQR* interquartile range, *SD* standard deviation, *T0* baseline assessment, *T1* follow-up assessment

^a^Contralateral capsular thickness was unavailable for one participant; this participant met the absolute criterion because affected-side capsular thickness was greater than 4 mm

Movement-evoked pain decreased by a median of 4–5 points across flexion, abduction, and external rotation (all *p* < 0.001; *r* = 0.81–0.86), whereas pain at rest did not change significantly (Table 2). Active and passive ROM improved significantly in all assessed planes (median changes, 15–57.5°; all *p* ≤ 0.001), with larger gains in passive ROM. Strength also improved in all three movement planes. The SF-12 Physical Component Summary improved and the DASH score decreased, whereas muscle tone and the SF-12 Mental Component Summary did not change significantly.

**Table 2:** Changes in clinical outcomes from T0 to T1

Clinical Outcome	T0 median (IQR)	T1 median (IQR)	Δ median (IQR)	*p* value	Effect size r
Pain at rest (NRS)	0 (0–0)	0 (0–0)	0 (0–0)	0.10	0.37
Pain – External rotation (NRS)	8 (8–9)	3.5 (2–6)	− 5 (− 6.25– − 3)	< 0.001	0.86
Pain – Abduction (NRS)	8 (8–9)	3.5 (2–6)	− 4 (− 6.25– − 2.5)	< 0.001	0.86
Pain – Flexion (NRS)	8 (6.75–8.25)	3 (1.75–5)	− 4 (− 6– − 2.5)	< 0.001	0.81
Passive ROM – External rotation (deg)	10 (3.75–30)	45 (28.8–70)	25 (20–41.2)	< 0.001	0.87
Passive ROM – Abduction (deg)	80 (70–90)	120 (100–162)	57.5 (10–80)	< 0.001	0.80
Passive ROM – Flexion (deg)	90 (77.5–92.5)	140 (109–156)	50 (18.8–75)	< 0.001	0.84
Active ROM – External rotation (deg)	0 (0–10)	20 (0–40)	15 (0–22.5)	< 0.001	0.74
Active ROM – Abduction (deg)	30 (17.5–72.5)	95 (47.5–130)	40 (0–85)	0.001	0.73
Active ROM – Flexion (deg)	25 (0–72.5)	110 (63.8–130)	47.5 (16.2–86.2)	< 0.001	0.79
External rotation strength (MRC)	2.5 (0.75–3)	3 (2–4)	0.25 (0–1)	0.010	0.58
Abduction strength (MRC)	2.5 (1–3)	3.25 (2.75–4)	0.75 (0–1.12)	0.010	0.56
Flexion strength (MRC)	2.5 (0.75–3)	3.25 (3–4)	1 (0–1)	0.004	0.64
Muscle tone – External rotation (MAS)	1.25 (0–2)	1 (0–3)	0 (0–1)	0.10	0.34
Muscle tone – Abduction (MAS)	1 (0–2)	1 (0–2)	0 (0–0)	0.90	0.02
Muscle tone – Flexion (MAS)	0 (0–2)	0 (0–2)	0 (0–0)	0.90	0.02
SF-12 Physical Component Summary	20 (16–25.5)	32.5 (22.2–42.2)	7.5 (0–15.8)	< 0.001	0.74
SF-12 Mental Component Summary	50 (45.8–51.2)	52.5 (47–55.5)	2 (0–8.25)	0.20	0.31
DASH score	93 (72.5–100)	77.5 (57.5–100)	0 (− 20.5–0)	0.030	0.48

*DASH* Disabilities of the Arm, Shoulder and Hand, *IQR* interquartile range, *MAS* Modified Ashworth Scale, *MRC* Medical Research Council, *NRS* Numeric Rating Scale, *ROM* range of motion, *SF-12* 12-item Short Form Health Survey

**Fig. 3:**
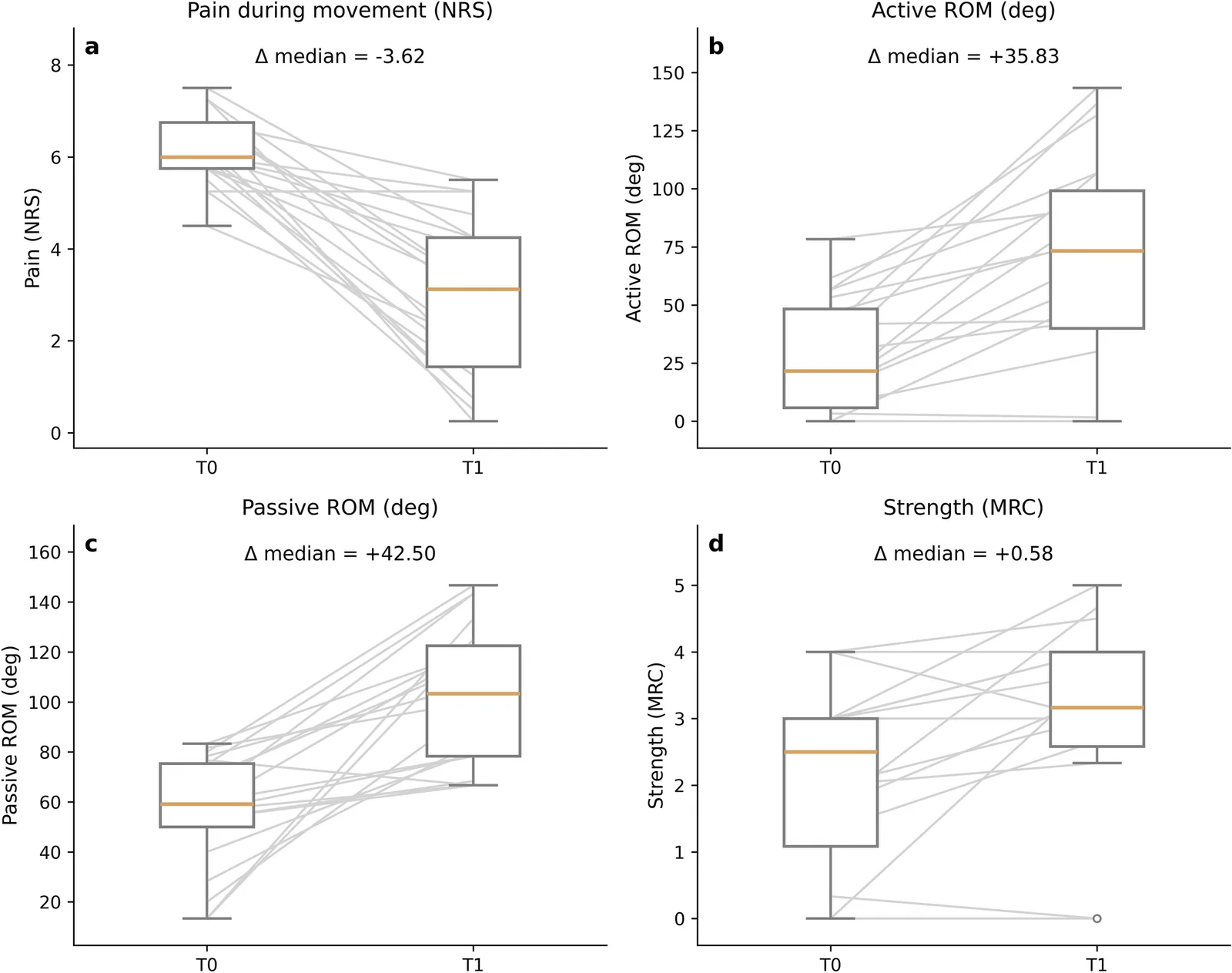
Domain-level clinical changes from baseline (T0) to follow-up (T1). Each line represents one participant; boxplots show medians and interquartile ranges. **A** Movement-evoked pain; **B** active ROM; **C** passive ROM; **D** strength

Figure 3 summarizes participant-level changes in movement-evoked pain, active ROM, passive ROM, and strength.

### Ultrasound outcomes

The proportion of participants meeting the prespecified ultrasonographic criteria for adhesive capsulitis decreased from 20/20 at T0 to 4/20 at T1 (exact McNemar *p* < 0.001), with 16 participants no longer meeting these criteria at follow-up.

Median affected-side axillary-pouch capsular thickness decreased from 3.7 mm (IQR, 3.1–4.7) to 2.45 mm (IQR, 2.0–3.2), with a median within-participant change of −1.4 mm (IQR, −2.3 to −0.7; *p* < 0.001; *r* = 0.85). No significant changes were observed in rotator cuff tendinopathy or tear scores, glenohumeral effusion, subacromial-subdeltoid bursitis, or long-head biceps tendon sheath effusion (Table 3).

**Table 3:** Changes in ultrasonographic outcomes from T0 to T1

Outcome	T0	T1	Change	*p* value	Effect size r
Adhesive capsulitis (0/1)	20/20 (100.0%)	4/20 (20.0%)	+ 0 new / -16 resolved	< 0.001	-
Affected-side capsular thickness (mm)	3.7 (3.1–4.7)	2.45 (2.0–3.2)	-1.4 (-2.3 to -0.7)	< 0.001	0.85
Rotator cuff tear score	1 (0–1)	1 (0–2)	0 (0–0.25)	0.10	0.37
GH joint effusion (0/1)	0/20 (0.0%)	4/20 (20.0%)	+ 4 new / -0 resolved	0.13	-
Rotator cuff tendinopathy score	0 (0–0.25)	0 (0–0)	0 (0–0)	0.32	0.22
SASD bursitis (0/1)	6/20 (30.0%)	5/20 (25.0%)	+ 2 new / -3 resolved	1.00	-
LHBT sheath effusion (0/1)	5/20 (25.0%)	4/20 (20.0%)	+ 2 new / -3 resolved	1.00	-

*IQR* interquartile range, *LHBT* long-head biceps tendon, *SASD* subacromial-subdeltoid, *T0* baseline, *T1* follow-up

Capsular-thickness measurements showed excellent reproducibility. Inter-observer reliability was ICC(A,1) = 0.964 (95% CI, 0.93–0.98), with an SEM of 0.181 mm and an MDC95 of 0.502 mm. Intra-observer reliability was ICC(A,1) = 0.975 (95% CI, 0.95–0.99), with an SEM of 0.145 mm and an MDC95 of 0.402 mm. Diagnostic agreement was substantial for both inter- and intra-observer assessments (Cohen’s κ = 0.833; agreement = 92.5%).

### Associations between capsular thickness changes and clinical outcomes

No significant associations were identified between capsular thinning and changes in pain, ROM, strength, muscle tone, disability, or quality of life (Table 4). Correlation coefficients ranged from ρ =  − 0.35 to ρ = 0.25 (all *p* ≥ 0.13).

**Table 4:** Associations between changes in affected-side capsular thickness and clinical outcomes

Clinical outcome	rho	*p* value
Pain at rest (NRS)	−0.15	0.531
Pain—External rotation (NRS)	0.11	0.649
Pain—Abduction (NRS)	0.19	0.425
Pain—Flexion (NRS)	0.10	0.665
Passive ROM—External rotation (deg)	−0.05	0.824
Passive ROM—Abduction (deg)	−0.11	0.635
Passive ROM—Flexion (deg)	−0.27	0.253
Active ROM—External rotation (deg)	−0.18	0.445
Active ROM—Abduction (deg)	−0.13	0.583
Active ROM—Flexion (deg)	−0.10	0.667
External rotation strength (MRC)	−0.35	0.134
Abduction strength (MRC)	−0.31	0.188
Flexion strength (MRC)	−0.25	0.297
Muscle tone—External rotation (MAS)	0.01	0.978
Muscle tone—Abduction (MAS)	0.12	0.608
Muscle tone—Flexion (MAS)	0.25	0.287
SF-12 Physical Component Summary (PCS)	−0.02	0.922
SF-12 Mental Component Summary (MCS)	−0.02	0.947
DASH score	−0.10	0.662

*DASH* Disabilities of the Arm, Shoulder and Hand, *MAS* Modified Ashworth Scale, *MRC* Medical Research Council, *NRS* Numeric Rating Scale, *PCS* Physical Component Summary, *MCS* Mental Component Summary, *ROM* range of motion, *SF-12* 12-item Short Form Health Survey

## Discussion

This longitudinal observational study identified two parallel patterns following ultrasound-guided hydrodilation with corticosteroid injection in patients with post-stroke adhesive capsulitis: movement-evoked pain and shoulder mobility improved, while affected-side axillary-pouch capsular thickness decreased. However, the magnitude of capsular thinning was not significantly associated with the magnitude of change in any clinical outcome. These findings support ultrasound as a tool for documenting structural capsular changes while cautioning against interpreting capsular thickness as a direct surrogate for symptomatic or functional recovery.

The post-treatment reduction in axillary pouch thickness aligns with recent literature showing capsular thinning after ultrasound-guided hydrodilation in primary adhesive capsulitis [22] and extends this observation to patients with post-stroke adhesive capsulitis. In the present cohort, median capsular thickness decreased by 1.4 mm, a change that exceeded the estimated MDC95 for both inter- and intra-observer measurements. Moreover, 16 of 20 participants no longer met the study ultrasonographic criteria for adhesive capsulitis at follow-up. These findings indicate that the observed reduction was greater than expected measurement error and suggest that capsular morphology may change following treatment even in the presence of neurological impairment. Nevertheless, the uncontrolled study design does not allow the relative contributions of hydrodilation, corticosteroid injection, concurrent rehabilitation, and the natural evolution of adhesive capsulitis to be distinguished.

The clinical pattern was also informative. Improvement was pronounced for pain provoked by passive movement, whereas pain at rest was minimal at baseline and remained unchanged. Gains in passive ROM exceeded those in active ROM, which may reflect relief of a mechanical capsular constraint while muscle weakness and impaired motor control continued to limit active movement after stroke. This interpretation is consistent with previous arthrographic and biomechanical findings in painful hemiplegic shoulders [1,11,12]. Improved passive mobility remains clinically relevant even without equivalent active recovery because it may facilitate positioning, hygiene, dressing, and participation in rehabilitation.

The lack of significant associations between structural and clinical changes should not be interpreted as evidence of a true dissociation. With only 20 participants, the exploratory correlation analyses had limited power to detect modest associations, and the restricted range of some outcomes further reduced sensitivity. Moreover, capsular thickness captures morphology rather than capsular compliance, inflammation, pain processing, or motor capacity. Associations between capsular thickness and clinical impairment observed at individual assessment timepoints may therefore not translate into parallel within-participant changes over time. Post-stroke shoulder pain reflects the interaction of peripheral and neurological mechanisms, including soft-tissue pathology, sensory dysfunction, abnormal tone and motor control, and central pain modulation [29–33]. Treatments targeting inflammation or nociceptive pathways may consequently improve pain without producing proportionate changes in capsular morphology [34]. Clinical recovery would therefore not be expected to track a single imaging measure linearly.

These findings have practical implications for ultrasound follow-up. Serial measurement of axillary-pouch thickness may provide an objective means of documenting capsular morphological changes over time and may complement clinical examination when passive ROM is difficult to interpret because of pain, spasticity, or impaired cooperation. However, treatment decisions should remain anchored to patient-relevant outcomes, particularly movement-evoked pain, passive function of the affected limb, and individual rehabilitation goals. Capsular thickness should not currently be used as a stand-alone marker of treatment success.

Several limitations should be considered when interpreting these findings. The absence of a control group limits the ability to distinguish the specific contribution of hydrodilation from that of corticosteroid injection, concurrent physiotherapy, natural neurological recovery, and the evolution of adhesive capsulitis. The relatively small and single-center sample limit the precision and generalizability of the findings. Finally, the use of a single follow-up assessment prevented characterization of the temporal trajectories of clinical and structural recovery.

Future controlled multicenter studies with larger samples, standardized treatment protocols and multiple follow-up assessments over longer periods are warranted to determine the specific contribution of hydrodilation and confirm the present findings.

## Conclusions

In patients with post-stroke adhesive capsulitis, ultrasound-guided hydrodilation with corticosteroid injection, delivered alongside neurorehabilitation, was followed by reduced movement-evoked pain, improved shoulder mobility, and measurable thinning of the affected axillary-pouch capsule. The magnitude of capsular thinning was not significantly associated with the magnitude of clinical improvement, supporting its interpretation as a complementary structural marker rather than a surrogate for recovery. Controlled studies are required before the observed clinical and structural changes can be attributed specifically to hydrodilation.

## Data Availability

The datasets generated and analyzed during the current study are not publicly available because they contain potentially sensitive clinical information. Anonymized data may be made available by the corresponding author upon reasonable request and subject to approval in accordance with applicable ethical and data-protection requirements.
